# Environmentally friendly silver nanoparticles synthesized from *Verbascum nudatum var.* extract and evaluation of its versatile biological properties and dye degradation activity

**DOI:** 10.1007/s11356-024-33424-5

**Published:** 2024-04-29

**Authors:** Ömer Hazman, Gofur Khamidov, Mustafa Abdullah Yilmaz, Mehmet Fatih Bozkurt, Mustafa Kargioğlu, Davlat Tukhtaev, Ibrahim Erol

**Affiliations:** 1 Department of Chemistry, Faculty of Science and Arts, Afyon Kocatepe University, 03200 Afyonkarahisar, Turkey; 2 Department of Organic Synthesis and Bioorganic Chemistry, Institute of Biochemistry, Samarkand State University, University Blvd-15, Samarkand, Uzbekistan; 3 Science and Technology Research and Application Center, Dicle University, 21280 Diyarbakır, Turkey; 4 Department of Analytical Chemistry, Faculty of Pharmacy, Dicle University, 21280 Diyarbakır, Turkey; 5 Faculty of Veterinary Medicine, Afyon Kocatepe University, 03200 Afyonkarahisar, Turkey; 6 Faculty of Science and Arts, Molecular Biology and Genetics, Afyon Kocatepe University, 03200 Afyonkarahisar, Turkey; 7 Department of Polymer Chemistry and Chemical Technology, Institute of Biochemistry, Samarkand State University, University Blvd-15, Samarkand, Uzbekistan

**Keywords:** Ag nanoparticle, Hydrothermal method, Green synthesis, Dye degradation, Biological properties

## Abstract

In the present study, green synthesis of silver nanoparticles (VNE-AgNPs) via *Verbascum nudatum* extract was carried out for the first time. The synthesized AgNPs were characterized by different spectral methods such as UV–vis, FTIR, XRD, TEM, and EDAX. According to TEM analyses, the average size range of AgNPs was 17–21 nm, and the dominant peaks in the 111°, 200°, 221°, and 311° planes in the XRD pattern indicated the Ag-NPs FCC crystal structure. FTIR data showed that VNE-AgNPs interacted with many reducing, capping, and stabilizing phytochemicals during green synthesis. VNE-AgNPs had higher antibacterial activity against *S. aureus* and *E. coli* bacterial strains with a maximum inhibition zone of 21 and 18 mm, respectively, than penicillin 5 IU, used as a positive control in the study. The cytotoxic effect of VNE-AgNPs appeared at a concentration of 50 µg/mL in L929 cells and 5 µg/mL in cancer (A549) cells. When the impact of VNE-AgNPs and C-AgNPs on inflammation was compared, it was found that VNE-AgNPs increased TNF-α levels (333.45 ± 67.20 ng/mg-protein) statistically (*p* < 0.05) more than TNF-α levels (256.92 ± 27.88 ng/mg-protein) in cells treated with C-AgNPs. VNE-Ag-NPs were found to have a degradation efficiency of 65% against methylene blue (MB) dye within 3 h.

**Graphical Abstract:**

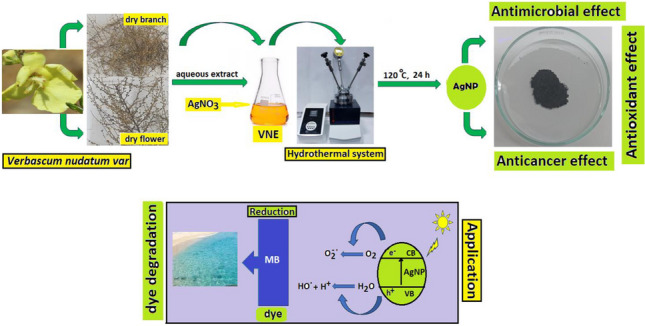

**Supplementary Information:**

The online version contains supplementary material available at 10.1007/s11356-024-33424-5.

## Introduction

AgNPs prepared by biosynthesis possess many essential properties due to their inherent environmentally friendly natural components and various bio-applications (Sharifi-Rad et al. 2024). Green synthesized AgNPs are used to produce antibacterial materials and wound healing creams and in applications in medicine, therapeutics, agriculture, diagnostics, etc. (Naghmachi et al. 2022; Nova et al. 2021). The antimicrobial activity of AgNPs is attributed to the fact that Ag^+^ ions released into the environment pass through the membrane and cause cell death. However, the dose-dependent toxicity of AgNPs poses a severe problem in terms of living health. Recent studies have shown that biosynthesized AgNPs have lower cytotoxicity than those synthesized by chemical methods (Gong et al. 2024; Kim et al. 2024). Ag NPs can be used in water treatment due to their superior properties, such as high antimicrobial activity, broad-spectrum pollutant removal capacity, low toxicity, and reusability.

The antimicrobial properties of AgNPs can be used to destroy microorganisms such as bacteria, viruses, and fungi in water sources. In addition, the catalytic property of AgNPs can be used to break down organic pollutants in water resources (Shruti et al. 2024; Yaglioglu et al. 2022). In this way, water resources pollution can be reduced, and water quality can be improved (Skiba et al. 2019). Research on the use of AgNPs to remove water pollution is ongoing. With the advancement of research in this field, it may be possible to use AgNPs as a more effective and safe method to remove water pollution (Madhu et al. 2022).

The common mullein (*Verbascum nudatum var. nudatum*) is an endemic species from the Scrophulariaceae family. It is distributed only in the Antalya subregion in Turkey. It is scientifically defined as *Verbascum nudatum var. nudatum Murb.* The name *Verbascum* mullein is probably derived from the Latin word barbascum or barba, meaning beard. In studies conducted on many *Verbascum* species, it has been reported that the plants have diuretic, diaphoretic, and sedative properties thanks to secondary metabolites such as alkaloids, saponins, flavonoids, lactones, coumarins, and ascorbic acids found in their extracts (Kaur and Upadhyaya 2016; Riahi and Ghahremaninejad 2019; Georgiev et al. 2011).

This study primarily focused on investigating the potential of the aqueous extract of *Verbascum nudatum var. Nudatum* to produce AgNPs by component analysis. The biological properties of the characterized AgNPs, such as antimicrobial, anticancer, and antioxidant, were compared with chemically synthesized AgNPs (C-AgNPs). In addition, the photocatalytic effect of the produced VNE-AgNPs against methylene blue (MB) dye was investigated. The effect of photo agents retained during biosynthesis on the biological and photocatalytic properties of biosynthesized AgNPs was also examined.

## Materials and methods

Information on the synthesis methods and measurements used in the study can be found in the supporting information file.

## Results and discussion

### Characterization of VNE-AgNPs

**Fig. 1:**
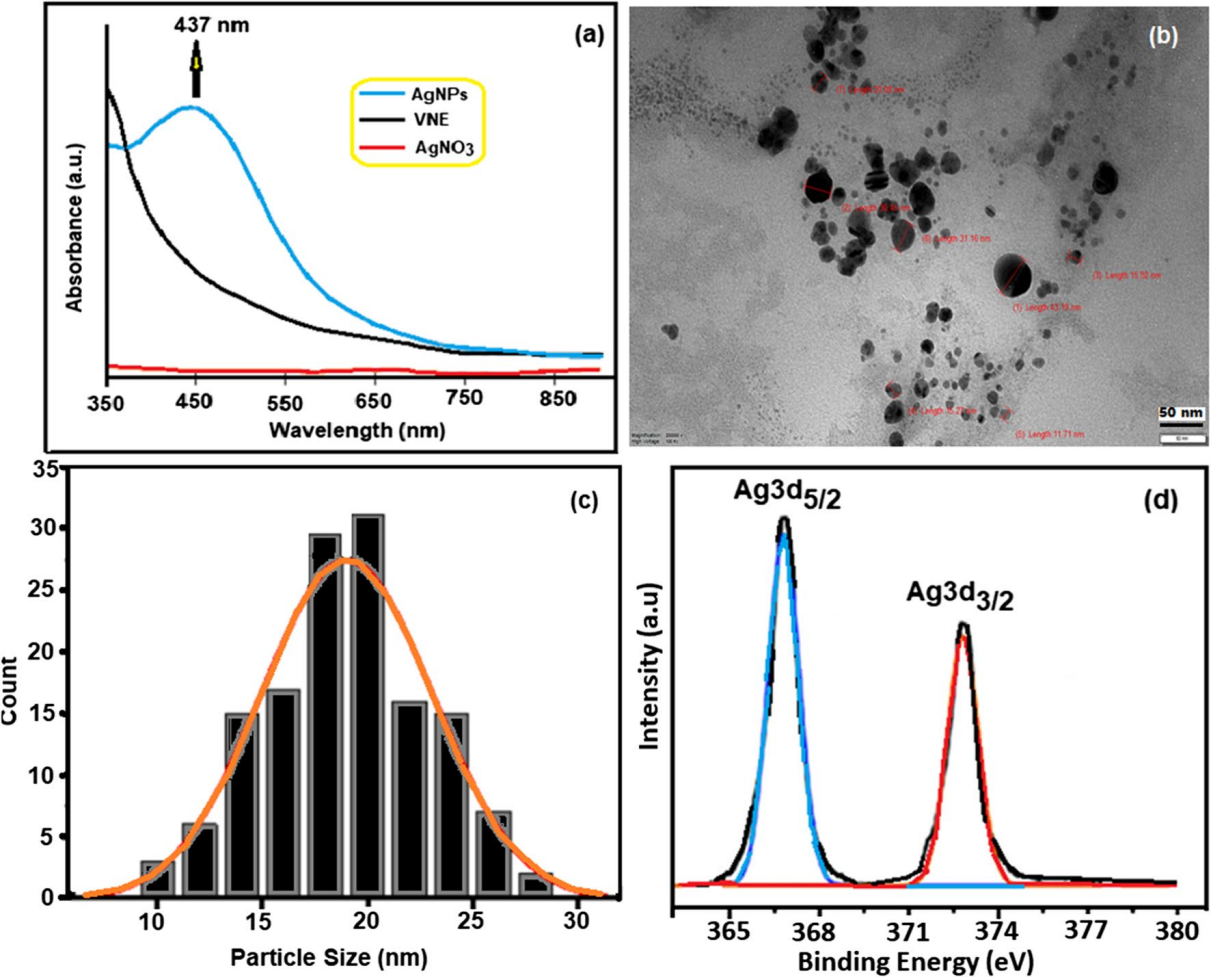
**a** UV–vis spectrum of AgNPs, VNE, and AgNO_3_; **b** TEM image of VNE-AgNPs; **c** particle size distribution of VNE-AgNPs; **d** XPS analysis of VNE-AgNPs

The synthesis of AgNPs was visually evident from the color change of the VNE. The color, brown at the beginning, changed to dark green after the process. Furthermore, after the hydrothermal treatment, the mixture was poured into petri dishes, and after drying in the open air, silver mirrors were visible. UV–vis spectroscopy of the synthesized AgNPs and the starting material AgNO_3_ and VNE are shown in Fig. 1a. The SPR peak of the VNE-AgNPs at 433 nm confirmed that the green synthesis was successful.

TEM micrographs of VNE-AgNPs presented in Fig. 1b show that they have a spherical shape. In addition, it is understood from the histogram illustrated in Fig. 1c that the average particle size of AgNPs is mainly 17–21 nm. From the XPS analyses of the synthesized VNE-AgNPs presented in Fig. 1d, the binding energy values for Ag3d_5/2_ and Ag3d_3/2_ were 366 and 372 eV, respectively.

**Fig. 2:**
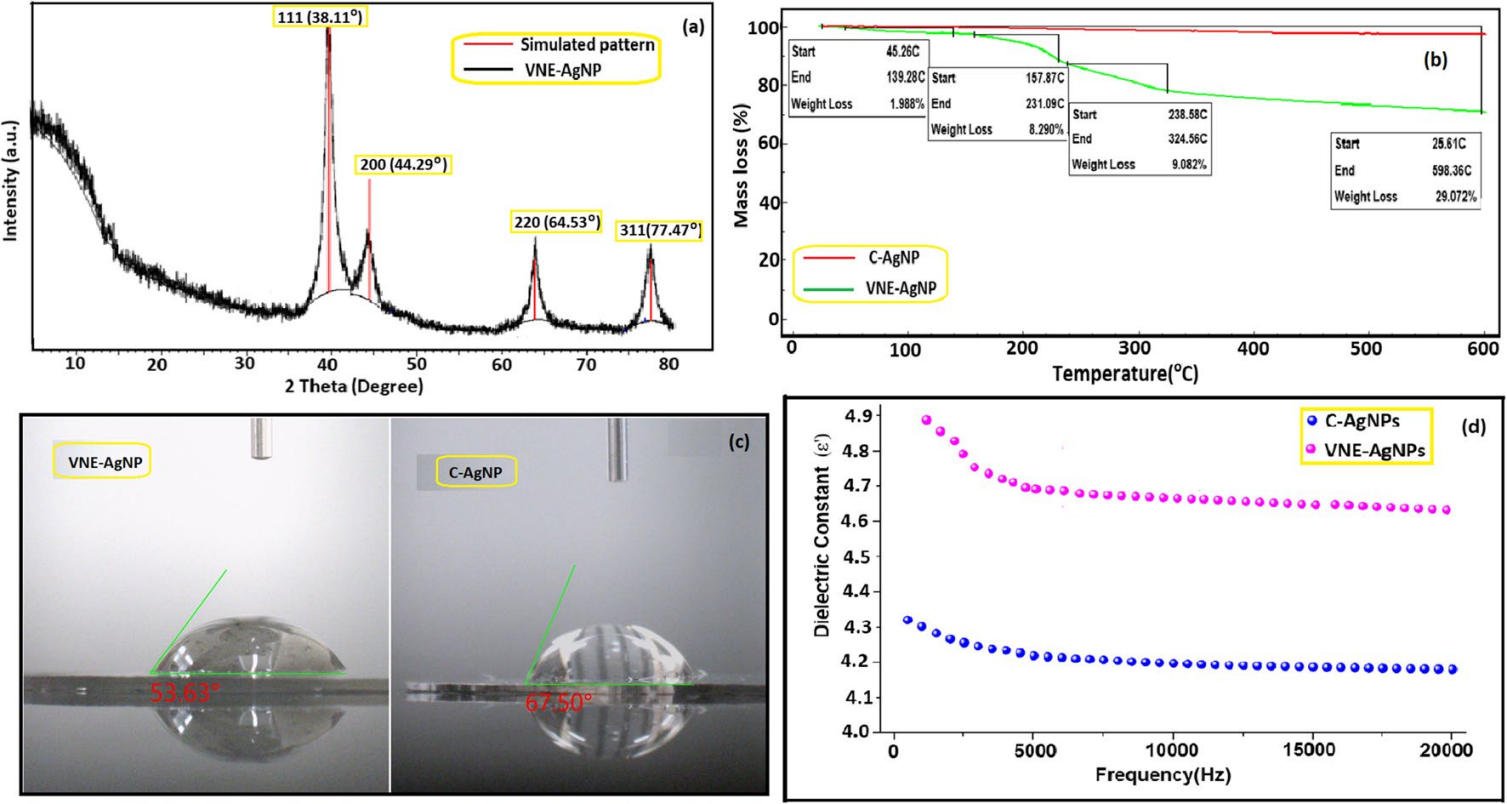
**a** XRD of VIE AgNPs; **b** TGA of VIE-AgNPs and C-AgNPs (heating rate, 20 °C/min, in N_2_); **c** contact angle of VNE-AgNPs and C-AgNPs; **d** variation of the dielectric constant of AgNPs with frequency at 2 kHz and 25 °C

Figure 2a shows the XRD patterns of VNE-AgNPs, including Bragg reflections in the range 20° ≤ 2 θ ≤ 80°. The position and width of XRD peaks can give information about the average size and shape of nanoparticles. This information can help to optimize the synthesis and processing conditions of nanoparticles. XRD patterns of AgNPs can be used to understand their physical and chemical properties and to identify the different phases present in the nanoparticles. The diffraction patterns of VNE-AgNPs observed at 38.11°, 44.29°, 64.53°, and 77.47° show sharp and intense peaks corresponding to the (111), (200), (220), and (311) planes, confirming the surface finned cubic (FCC) structure (JCPDS, card No. 04–0783) (Hong et al. 2022). The XRD data do not contain specific peaks of AgNO_3_ and suggest that the silver ions are entirely reduced to the corresponding metallic form. The average crystallite diameter of VNE-Ag NPs, with the help of the Debye–Scherrer formula, was found to be ≈ 22 nm.

TGA is a widely used method for determining the thermal properties of materials (Erol and Sahin 2015). TGA data obtained in the range of 25–600 °C were used to compare the thermal properties of synthesized VNE-AgNPs with commercial C-AgNPs and to investigate the effect of biosynthesis. In the thermogram in Fig. 2b, C-AgNPs showed no mass loss during the thermal process, while VNE-AgNPs suffered the mass loss in three different temperature ranges. A mass loss of approximately 2% was observed in the first interval between 45 and 139 °C. The possible cause of this loss may be surface water. The second mass loss was 8.2% in the 157–231 °C range, and the last mass loss was 9% in the 238–324 °C range. The total mass loss of VNE-AgNP in the 25–600 °C range was approximately 29%. It can be said that the mass losses other than the removal of surface water are related to the plant secondary metabolites retained on the surface of VNE-AgNPs during biosynthesis (Chinnasamy et al. 2023). Another data confirming this proposition is the contact angle values of AgNPs. As shown in Fig. 2c, the contact angle of C-AgNP is 67.50°, while that of VNE-AgNP is 53.63°. The fact that the surface of VNE-AgNPs is more hydrophilic than C-AgNPs may be due to the secondary metabolites containing polar functional groups, such as amide, ester, and hydroxyl, which were detected in the plant content. Molecules containing such groups cause the surface to be significantly hydrophilic.

The binding of VNE-derived herbal agents to AgNPs during biosynthesis was also evident from the dielectric measurements presented in Fig. 2d. While the dielectric constant of C-AgNPs was 4.12, it increased to 4.62 for VNE-AgNPs. The rotational motion of polar molecules in a dielectric material at maximum frequencies does not change rapidly with the applied field to reach equilibrium. The reason for this behavior is polarization effects due to the inability of dipoles to follow field changes at high frequencies. In insulators, electrons hardly move from one molecule to another, whereas in insulators, this electron remains bound to the atom. In this context, the observed change in the dielectric constant of VNE-AgNPs may be the orientation of the rising dipoles caused by the polar groups originating from the plant agents (Erol et al. 2022).

Fig. S1 shows the FESEM images at various magnifications used to investigate the structural, morphological, and surface properties of VNE-AgNPs. FESEM images illustrated that VNE-AgNPs have a regular and oval-spherical shape with an average size of 21 nm. The crystallite size obtained by XRD and TEM almost matched the particle size measured by FESEM. In addition, the EDS-MAP analysis of VNE-AgNPs presented in Fig. S2 exhibits a homogeneous composition, and the elemental analysis results confirm the existence of pure AgNPs.

**Fig. 3:**
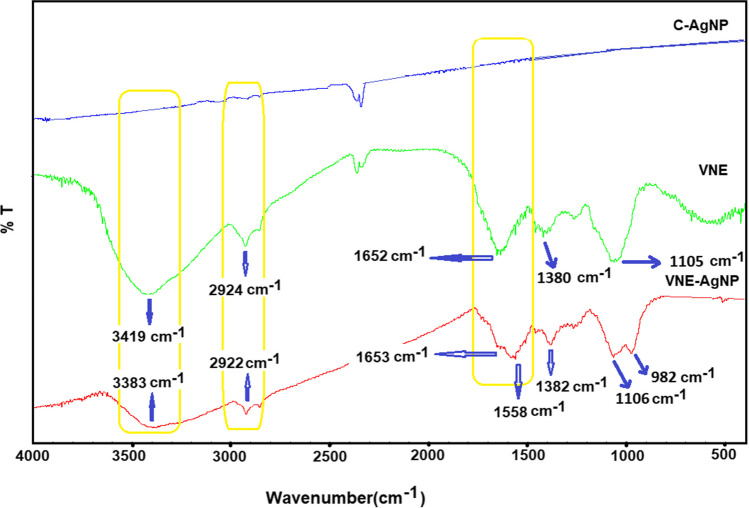
FTIR spectra of VNE, VNE-AgNPs, and C-AgNPs (by KBr disc)

The FTIR spectrum can be used to determine the properties of the biological material and nanoparticles used to synthesize nanoparticles. The FTIR spectrum of biosynthesized VNE-AgNPs is presented in Fig. 3. C-AgNPs did not show any significant peak in FTIR. On the other hand, the FTIR spectrum of VNE contained a wide range of signals identifying many functional groups. The broad and diffuse band at 3419 cm^−1^ represents OH, NH, and NH_2_ groups. These functional groups correspond to amine, alcohol, and phenolic compounds in plant extracts. The band at 2924 cm^−1^ is related to aliphatic C-H stretching vibrations. These bands may be due to ringed or straight-chain aliphatic structures in secondary metabolites. The broadening of the band at 1652 cm^−1^ suggests the presence of carbonyl group-containing compounds such as amides, ketones, and aldehydes. These bands may be sourced from biological molecules such as proteins and peptides. Strain vibration bands related to C-O bonds were observed at 1380 cm^−1^. The bands at 1105 cm^−1^ can be assigned to the strain vibrations of CH_2_’s in the main chain of secondary metabolites and ring systems (Gabriela et al. 2017). These bands could be from the sugars.

The peaks observed in the FTIR spectrum of VNE-AgNPs indicate the presence of functional groups belonging to phyto-agents, which play a role in capping and stabilizing the nanoparticles. The presence of these peaks indicates that the surfaces of AgNPs are coated, and oxidation is prevented. The intensity of the absorption band at 3383 cm^−1^ decreased compared to VNE’s. This data indicates that OH groups in reducing phenolic molecules participate in a redox reaction reducing Ag^+^ ions (Thi et al. 2022). The bands at 2922 cm^−1^ can be attributed to the C-H stretching vibration of hydrocarbon groups. The broadened band at 1653 cm^**−**1^ belongs to metabolites containing many carbonyl (C = O) functional groups (Maheshwaran et al. 2020). The bands observed at 1106 cm^−1^ are attributed to alkoxy (R-O) groups, and the band at 982 cm^−1^ is attributed to aliphatic C-H deformation. As can be seen from the bands labeled on the FTIR spectrum, secondary metabolites such as protein, carbohydrate, and sugar are retained by AgNPs.

### Total phenolic content and phytochemical profile of VNE

No comprehensive research on *Verbascum nudatum* species was found when the literature was examined. From this point of view, the species’ phytochemical profile and chemical and biological properties were investigated for the first time in the present study. When the total phenolic content analysis data using 1 mg each of caffeic acid and VNE extract were analyzed, it was determined that the entire phenolic content of VNE aqueous extract was 113.8 ± 19.8 µgGallicAcidEqiuvalent/mg-extract (Fig. S3a). When caffeic acid, a phenolic substance, is compared with total phenolic content (880.4 ± 62.4 µgGallicAcidEqiuvalent/mg-extract), this ratio seems low. However, considering that caffeic acid is a phenolic standard, it is expected that the total phenolic matter levels of caffeic acid analyzed by weighing 1000 µg are close to 1000 µgGallicAcidEqiuvalent/mg-extract levels, and it is an indication that the analysis was performed correctly. Considering that there may be many substances, such as water-soluble organic acids, alkaloids, and glycosides other than phenolic acids in VNE content, it can be viewed as a reasonable situation that 1000 µg extract contains approximately 11.4% phenolic substances. This ratio was acceptable when compared with the studies conducted in the literature with aqueous extracts of *Verbascum* species. It is reported in the literature that the phenolic content of *Verbascum* extracts obtained using water as a solvent can vary between 3.5 and 40% (Zengin et al 2021; Boğa et al. 2016).

The presence of the 53 components shown in Figure S4a and Table 1 in VNE was investigated quantitatively. While the presence of 16 components was quantitatively determined (Figure S4b), 34 components were not found in VNE. It is seen that quinic acid, gentisic acid, and protocatechuic acid are more in VNE than other components. When the studies conducted with *Verbascum* species whose phytochemical components were investigated (Boğa et al. 2016; Amini et al. 2022; Gupta et al. 2022; Tatlı and Akdemir 2004; Georgiev et al. 2011; El Gizawy 2019), it was determined that quinic acid, gentisic acid, and protocatechuic acid were absent or very low in other species. In this respect, *Verbascum* datum differs from other *Verbascum* species with its major components.

**Table 1:** Phytochemical profile of VNE

Component type*	Concentration (µg analyte/mg extract)
Quinic acid	1.669
Gentisic acid	1.475
Protocatechuic acid	0.591
Isoquercitrin	0.359
Caffeic acid	0.148
Quercetin	0.134
Luteolin	0.123
p-Coumaric acid	0.117
Salicylic acid	0.111
Chlorogenic acid	0.057
Protocatechuic aldehyde	0.052
Astragalin	0.031
Apigenin	0.01
Kaempferol	0.009
Genistein	0.006
Amento flavone	0.002

^*^Components whose presence cannot be determined in VNE: fumaricaid, cyranoside, cosmosiin, naringeninA, hesperetin, chrysin, aconitic acid, gallic acid, epigallocatechin, catechin, tannic acid, epigallocatechingallate, cynarin, 4-OH benzoic acid, epicatechin, vanilic acid, syringic acid, vanillin, syringicaldehyde, daidzin, epicatechingallate, piceid, ferulic acid, sinapic acid, coumarin, miquelianin, hesperidin, o-coumaric acid, genistin, rosmarinic acid, ellagic acid, quercitrin, nicotiflorin, fisetin, daidzein, salicylic acid, rutin

### Reduction mechanism and chemical interaction

Polyphenols form capsules for the growth of nanoparticles through intramolecular and intermolecular hydrogen bonding. Hydrogens in the -OH groups in polyphenol molecules form the Ag +  → Ag^0^ reaction template on these high-energy surfaces and act as a reducing agent, reducing it to metallic Ag. Due to the high density of hydroxyl groups, the reduction and transport of Ag^+^ ions is possible in cages with polar and hydrophilic interiors (Gautam et al. 2007).

In biosynthesis processes, secondary metabolites can attach to the surface of AgNPs. Weak electrostatic interactions such as Van der Waals forces play a role in this attachment. These forces can ensure weak binding of the secondary metabolite to the nanoparticle surface (Zulfiqar et al. 2024). In addition, chelation can occur between donor groups such as OH and NH_2_ of secondary metabolites and AgNPs via coordination bonds (Liu et al. 2023). Some secondary metabolites can function as low molecular weight polymers. These polymers can coat and stabilize the AgNP surface (Chen et al. 2020). These interactions help stabilize AgNPs by preventing aggregation and precipitation and may confer biological activities such as antibacterial, antifungal, antioxidant, or anticancer to the nanoparticle. Ultimately, the adhesion of secondary metabolites to the AgNP surface can significantly affect the properties and behavior of nanoparticles, affecting their potential for use in medical, agricultural, and environmental applications.

### DPPH radical scavenging activity

As seen in Fig. S3b, BHT is a synthetic antioxidant with the highest reduction potential in DPPH radical scavenging activity. However, especially at high concentrations (5–10 mg/mL), the DPPH inhibition of VNE-AgNPs was close to that of BHT and was found to be a more potent reductant than C-AgNPs. DPPH inhibition rates of VNE extract were lower. These data indicate that the bioactive groups shown by FTIR, contact angle, and dielectric constant measurements to be retained by VNE-AgNPs during green synthesis may be mostly antioxidant-effective components. Therefore, the DPPH radical scavenging activity of VNE-AgNPs may have increased. The DPPH radical scavenging activity of *Verbascum nudatum* was determined for the first time in this study. In studies conducted with other *Verbascum* species, it has been reported that as the concentrations of extracts of *Verbascum* species prepared with different solvents such as ethanol and water increase, an increase in DPPH radical scavenging activity is observed. The reason for the rise in DPPH reduction potential, depending on concentration, is shown to be secondary metabolites of phenolic/flavonoid structure in the extracts (Boğa et al. 2016). In this context, the results we obtained seem compatible with the literature.

### Antimicrobial activity

**Fig. 4:**
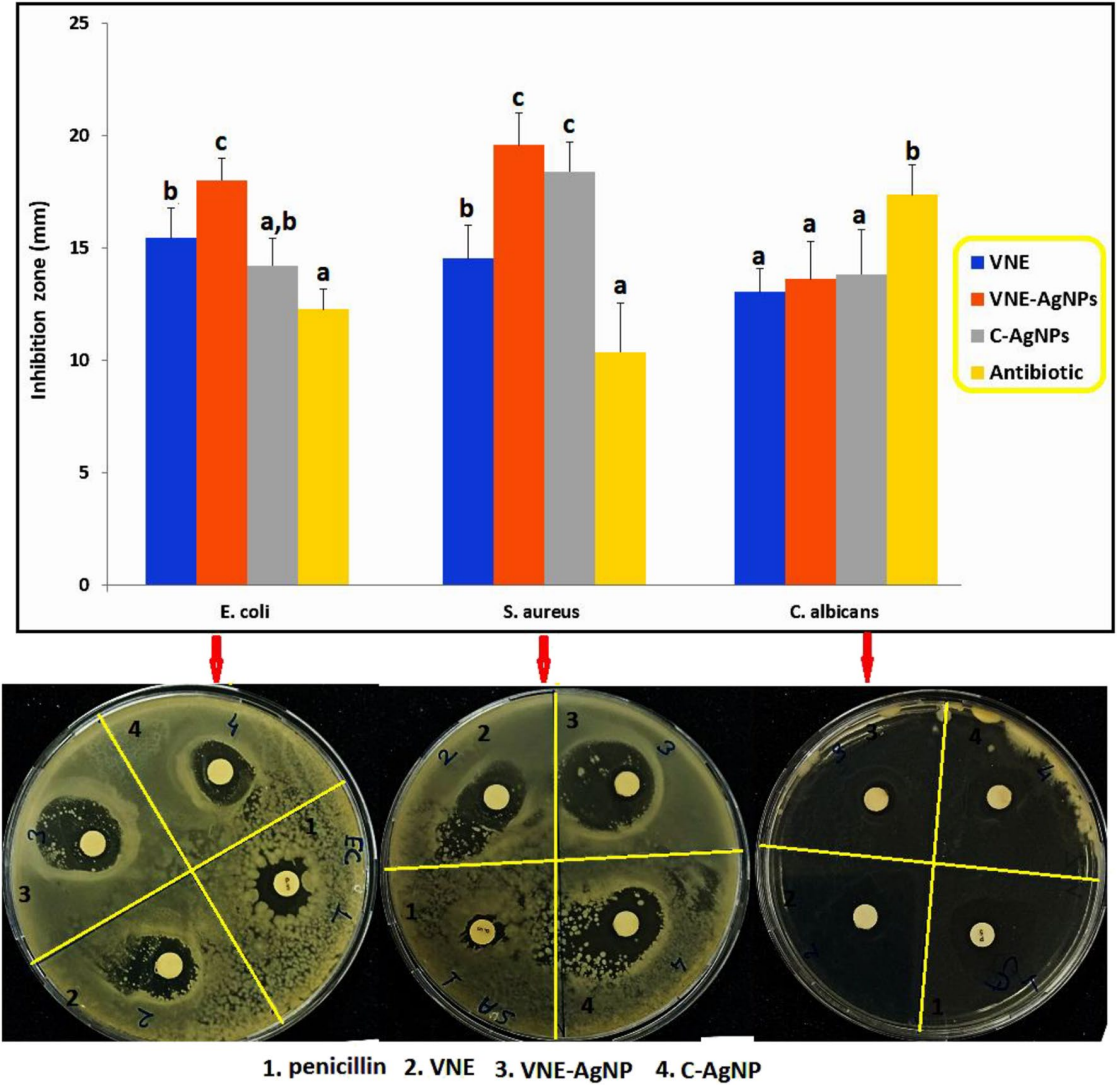
Antimicrobial activity of VNE and nanoparticles. Data are presented as mean ± standard deviation (*n* = 3). (a–c) There is a statistical difference between data belonging to the same microorganism strain and data with different superscripts (*p* < 0.05)

In the literature, the antimicrobial activities of different *Verbascum* species such as *Verbascum thapsus*, *Verbascum pinetorum*, *Verbascum lasianthum*, *Verbascum euphraticum*, and *Verbascum oocarpum* have been determined (Zengin et al. 2021; Boğa et al. 2016; El Gizawy et al 2019; Turker and Camper 2002). It was determined in this research that *Verbascum nudatum*, like the *Verbascum* species examined, has antimicrobial activity. When the data presented in Fig. 4 was examined, it was determined that the antimicrobial activity of VNE against *S. aureus* and *E. coli* bacterial strains was higher than the antibiotic (penicillin, 5 IU) used as a positive control in the study. On the other hand, analyses conducted with *C. albicans* show that the antifungal activity of VNE remains lower than its antibacterial activity. The number of studies using *Verbascum* species in nanoparticle synthesis is very few. When these studies (Bekru et al. 2022; Soto et al. 2022) are examined, it is seen that the antimicrobial activity of nanoparticles synthesized using *Verbascum* species has not been determined. However, in many studies with nanoparticles, it is reported that nanoparticles produced by chemical or green synthesis may show antimicrobial activity (Sivrier et al 2023; Erol et al. 2023). When the antimicrobial activity of VNE-AgNPs and C-AgNPs was compared in Fig. 4, it was determined that the effect of VNE-AgNPs against *E. coli* was statistically (*p* < 0.05) different and higher. When the results of the analyses with *S. aureus* and *C. albicans* strains were examined, it was observed that there was no significant difference between the activities of VNE-AgNPs and C-AgNPs.

### Cytotoxicity and anticarcinogenic activity

**Fig. 5:**
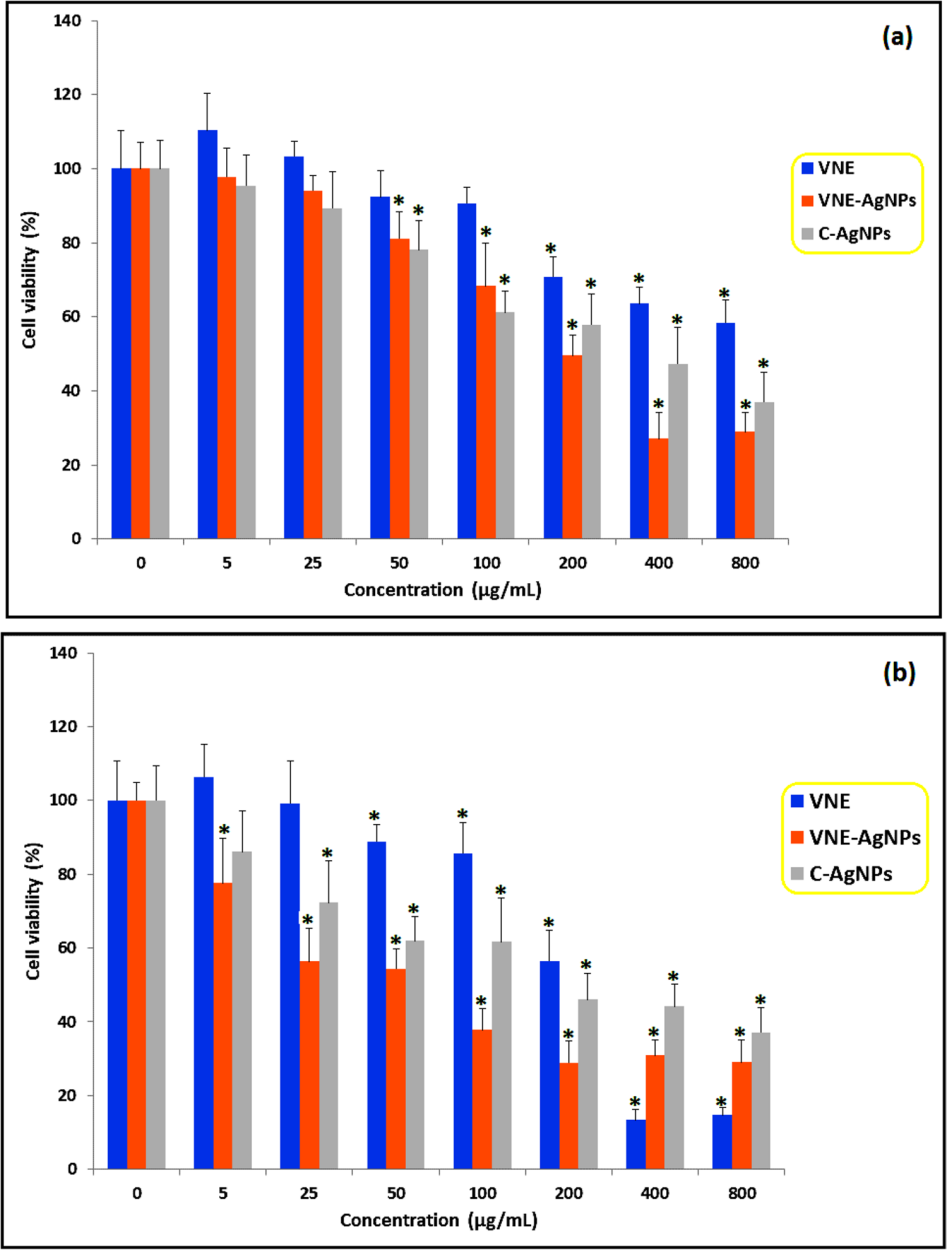
Cytotoxic and anticarcinogenic effects of VNE, C-AgNP, and C-VNE-AgNPs. **a** Cytotoxicity in L929 cells and **b** anticarcinogenic effect in A549 cells. *Indicates a statistical difference compared to the control group (*p* < 0.05)

The cytotoxicity of the samples was determined in a healthy cell line consisting of L929 cells (Fig. 5a). In contrast, the anticarcinogenic activity was determined in lung adenocarcinoma (A549) cells (Fig. 5b). VNE was not cytotoxic at low doses in healthy (L929) cells but showed a cytotoxic effect at 200 µg/mL and above doses. Considering that *Verbascum* species are used in diseases such as hemorrhoids, diarrhea, rheumatic pain, wounds, fungal infections, respiratory diseases, and skin complications due to inflammation (Boğa et al. 2016; Turker and Camper 2002), the lack of cytotoxicity of VNE at low doses in healthy cells suggests that it may be a reliable species for alternative medicine. Of course, further studies with different experimental models are needed to say this clearly. In A549 cells, cytotoxicity of VNE started at a concentration of 50 µg/mL and above, and its anticarcinogenic activity was extreme at doses of 200 µg/mL and above (Fig. 5b).

When the data presented in Fig. 5 are analyzed, the cytotoxic effect of VNE-AgNPs was observed at the lowest concentration of 50 µg/mL in L929 cells. In comparison, cytotoxic effects were determined at a 5 µg/mL concentration in cancer (A549) cells. The anticarcinogenic activity of C-AgNPs was observed at concentrations of 25 µg/mL and above. These data indicate that VNE-AgNPs have anticarcinogenic activity even at low concentrations and that this activity is higher than that of C-AgNPs at all doses. The probable reason for this may be related to the bioactive photo agents on the surface of VNE-AgNPs produced by green synthesis. The anticarcinogenic activities of plant species containing phenolic compounds such as quinic acid, gentisic acid, and protocatechuic acid, which are detected in VNE and whose amount is high, have been determined by different studies in the literature (Anoor et al 2022; Basit et al. 2023; Park et al. 2011).

The reason for the anticarcinogenic activity of VNE-AgNPs may be that the phytochemicals in VNE, which are reported to have anticarcinogenic effects, are retained on the nanoparticles as a result of green synthesis and released when applied to cells in the in vitro environment. There are very few studies in which *Verbascum* species were used in nanoparticle synthesis, and the anticarcinogenic effects of these nanoparticles were investigated. In one of these studies, the impact of gold nanoparticles obtained using *Verbascum thapsus* flower extracts and gold nanoparticles produced by the chemical method was analyzed. Similar to our study, it was reported that nanoparticles produced by green synthesis increased anticarcinogenic activity by retaining some bioactive components in the extract (Soto et al. 2022). The higher anticarcinogenic activity of VNE-AgNPs in A549 cells compared to chemically produced C-AgNPs is closely related to the particle size, morphology, and bioactive components retained on the surface. The particle size of VNE-AgNPs with higher anticarcinogenic activity in our study was smaller than C-AgNPs. In our research, the particle size of the chemically synthesized C-AgNPs obtained commercially (Sigma, cat. No: 576832) is reported to be < 100 nm (https://www.sigmaaldrich.com/TR/en/product/aldrich/576832), and the manufacturer sells AgNPs with smaller ( < 50 nm) particle size at higher prices. The particle distribution of the VIE-AgNPs synthesized in our study, determined by TEM and XRD, was mostly in the range of 15–25 nm, and all were below < 30 nm. These data suggest that particle size may be one of the reasons for the high cytotoxicity of VNE-AgNPs in A549 cells. In parallel with our findings, the literature also reports an inverse correlation between the particle size of nanoparticles and cytotoxicity (Park et al. 2011).

### Evaluation of the effect on oxidative stress, inflammation, and proliferation levels

As stated in the previous section, the particle size, morphology, and bioactive components retained on the surface of nanoparticles shape their cytotoxic activity in cancer cells by stimulating/suppressing signaling pathways related to oxidative stress, inflammation, inflammation, and apoptosis. There is no study conducted with *Verbascum nudatum* in this direction. However, gold nanoparticles synthesized using *Verbascum thapsus* extract, another *Verbascum* spp., have been reported to increase reactive oxygen species (ROS) formation and stimulate apoptosis in cancer cells (Soto et al. 2022). In our study, it was determined that oxidative and inflammation did not change statistically as a result of VNE application compared to the control group data. However, it is seen in Table 2 that when VNE-AgPs and C-AgNPs were applied to A549 cells at a cytotoxic dose, oxidative stress (TAC, TOC, OSI) and inflammation (TNF-α, IL-1β, and β-Defensin)-related parameter levels increased compared to the control group. In contrast, proliferation (Ki67 positive cells) levels were not affected. The studies conducted support our findings. It is stated that AgNPs increase oxidative stress by increasing ROS levels in A549 cells and may cause inflammation by increasing proinflammatory cytokine levels (Chairuangkitti et al. 2013; Matysiak-Kucharek et al. 2023).

**Table 2:** Effects of the materials used in the study on oxidative stress, inflammation, and proliferation in A549 cells

Parameters	Experimental groups				*p*
	Control	VNE	VNE-AgNPs	C-AgNPs	
TNF-α (ng/mg-protein)	86.76 ± 17.51^a^	89.69 ± 4.68^a^	333,45 ± 67.20^b^	256.92 ± 27.88^b^	0.002
IL-1β (pg/mg-protein)	2.11 ± 0.81^a^	2.70 ± 0.65^a^	41.18 ± 16.76^b^	48.34 ± 8.43^b^	0.013
DEF-β2 (ng/mg-protein)	539.69 ± 28.68^a^	606.19 ± 30.33^a^	2503.46 ± 452.60^b^	2361.44 ± 124.31^b^	0.000
TAC (mmol Equiv. Torolox /mg-protein)	0.70 ± 0.07^a^	0.65 ± 0.02^a^	1.50 ± 0.22^b^	1.56 ± 0.03^b^	0.000
TOC (µmol Equiv.H_2_O_2_/mg-protein)	59.08 ± 5.44^a^	64.12 ± 1.86^a^	211.29 ± 49.61^b^	168.64 ± 5.81^b^	0.000
OSI (AU)	84.56 ± 8.03^a^	97.90 ± 3.58^a,b^	139.37 ± 12.04^c^	108.13 ± 2.17^b^	0.000
Total protein (mg/mL)	1.83 ± 0.07^c^	1.67 ± 0.01^b^	0.53 ± 0.12^a^	0.67 ± 0.02^a^	0.000
Ki67 positive cells (%)	185.36 ± 3.37	185.48 ± 3.99	185.46 ± 6.89	184.79 ± 5.09	0.124
iNOS intensity (AU)	55.24 ± 3.12	57.21 ± 5.25	61.98 ± 3.80	62.37 ± 6.96	0.985

Abbreviations: *TGF-β1* tumor growth factor-1β, *TNF-α* tumor necrosis factor-α, *DEF-β2* β-defensin 2, *VNE Verbascum nudatum* extract, *VNE-AgNPs* silver nanoparticle, *C-AgNPs* produced using *Verbascum nudatum* aqueous extract, silver nanoparticle produced by chemical method

^*^Data are given as mean ± standard deviation (*n* = 3)

^a^^,^^b,c^The difference between the averages carrying different exponents in the same line (parameter) is statistically significant (*p* < 0.05)

**Fig. 6:**
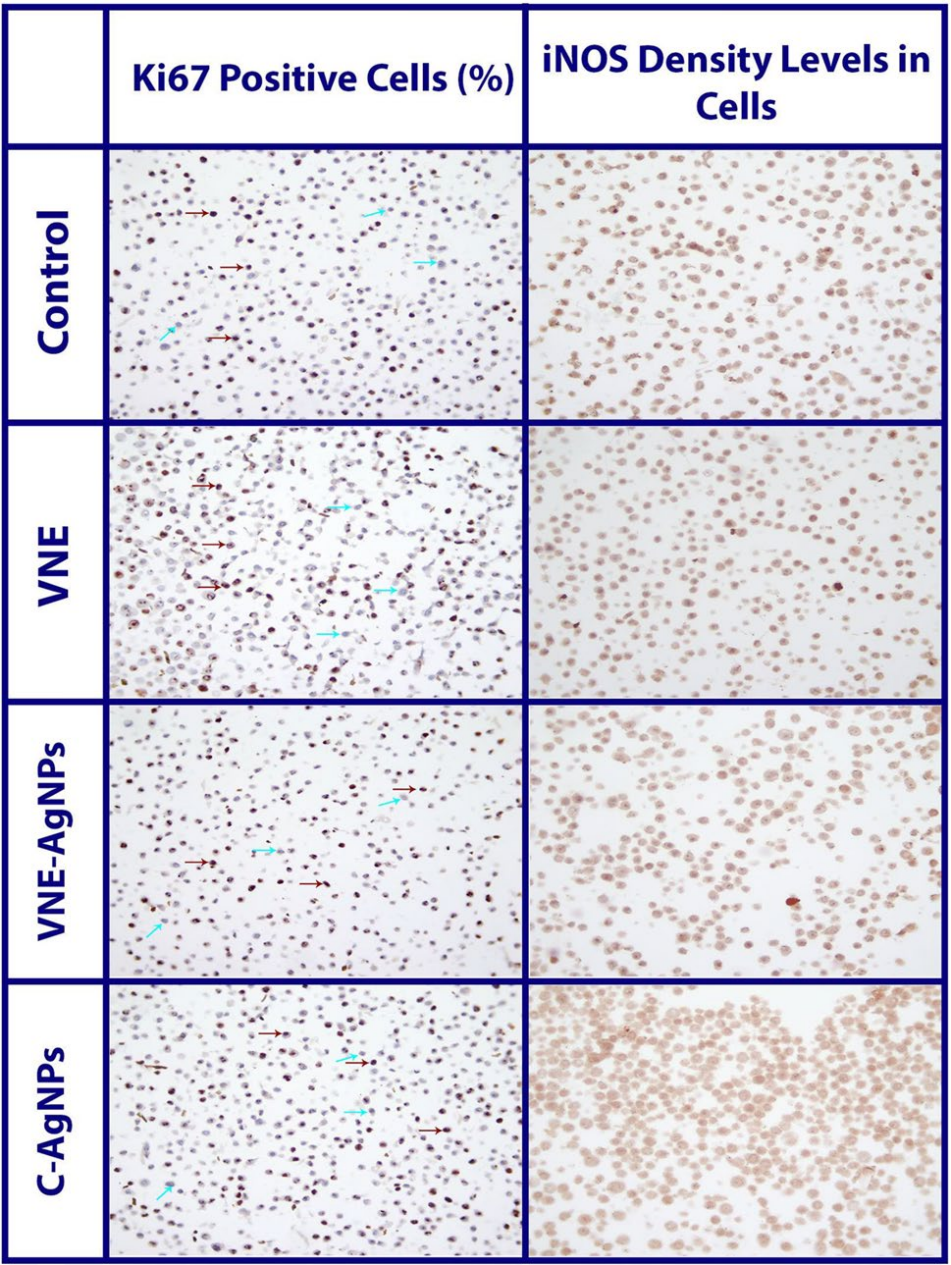
Microscope images of cells used in the evaluation of proliferation (Ki 67) and iNOS levels by immunohistochemical method

When the effect of VNE-AgNPs and C-AgNPs on inflammation was compared, it was determined that VNE-AgNPs increased TNF-α levels (333.45 ± 67.20 ng/mg-protein) statistically (*p* < 0.05) more than TNF-α levels (256.92 ± 27.88 ng/mg-protein) in C-AgNPs treated cells. There was no statistical difference between IL-1β and DEF-β2 levels in cells treated with both nanoparticles. Similarly, when the effects of nanoparticles on TAC, TOC, and iNOS levels (Table 2, Fig. 6), which are oxidative stress parameters, were evaluated, no statistical difference was observed. However, when OSI levels were analyzed (Table 2), it was observed that OSI levels (139.37 ± 12.04) were higher in VNE-treated cells. These data showed that VNE-AgNPs increased inflammation via TNF-α, a proinflammatory cytokine, and oxidative stress by increasing OSI levels more than C-AgNPs in A549 cells. These data can be shown as one of the reasons for the high anticarcinogenic activity of VNE-AgNPs in A549 cells, as stated in the previous section. Because the increase in oxidative stress and inflammation in cells treated with VNE-AgNPs stimulates apoptosis and causes an increase in anticarcinogenic activity, particle size can be shown as one of the reasons why VNE-AgNPs increase inflammation and oxidative stress more. Another reason may be related to the phytochemicals attached to the nanoparticle surface due to biosynthesis. It has been reported that bioactive components such as luteolin and chlorogenic acid contained in VNE may increase oxidative stress and inflammation in A549 cells (Zhao et al. 2011; Yamagata et al. 2018).

### Photocatalytic activity of AgNPs for dye degradation

In recent years, nano-sized materials and particles have been frequently used as catalysts for converting environmental pollutants into less harmful compounds.

**Fig. 7:**
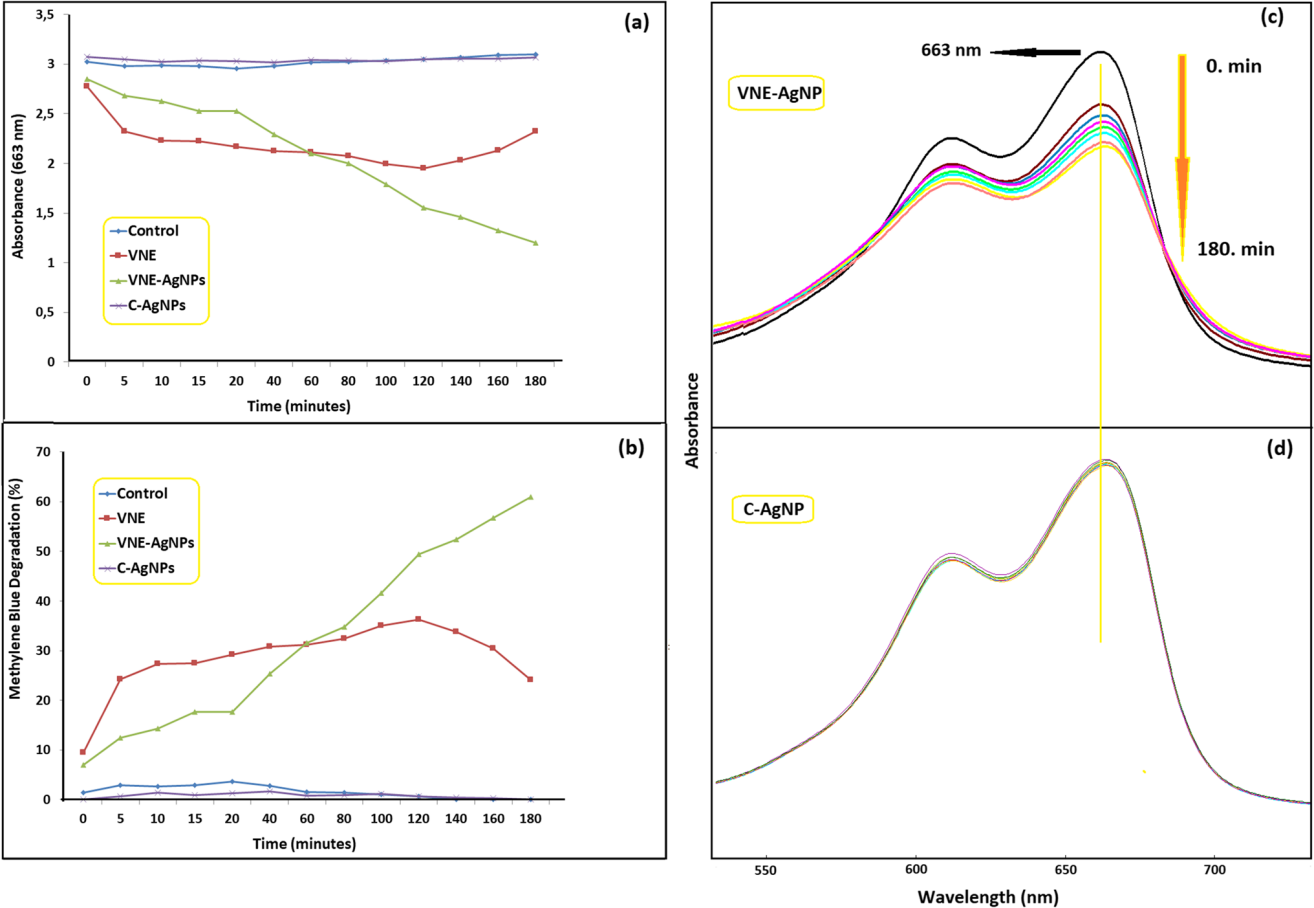
**a** Effect of VNE and nanoparticles on absorbance change after addition to MB solution, **b** effect of VNE and nanoparticles on MB % degradation rates depending on time, **c** UV–vis spectra of VNE-AgNP effective MB solutions at different times during the photodegradation process, and **d** UV–vis spectra of C-AgNP effective MB solutions at different times during the photodegradation process

In particular, metal oxide-based nanostructured photocatalysis is one of the promising approaches in this regard (Akkari et al. 2022). The number of studies investigating *Verbascum* spp. species in this regard is limited. One of these studies reported that CuO-ZnO hybrid nanoparticles produced by *Verbascum sinaiticum Benth* plant provided MB degradation within 120 min (Bekru et al. 2022; Yu et al.^2024^). In our study, we obtained VNE-AgNPs using *Verbascum nudatum*, another *Verbascum* spp. The MB degradation levels of the obtained VNE-AgNPs and the MB degradation levels of VNE and C-AgNPs were determined separately and compared. The MB degradation levels of the obtained VNE-AgNPs and the MB degradation levels of VNE and C-AgNPs were determined and compared separately. As shown in Fig. 7a, the absorption value of the peak at 663 nm decreased with time in MB solutions containing VNE-AgNPs. In contrast, no change was observed in solutions containing C-AgNPs. This result is seen in the UV spectra obtained at the total photodegradation time and presented in Fig. 7c and d. Another remarkable result was that the solutions containing VNE had an effective reduction potential of up to about 120 min. As a result of the decrease of the peak at 663 nm belonging to MB, it can be seen that the most significant reduction potential belongs to VNE-AgNPs, with 65% at the end of 180 min, as presented in Fig. 7b. It was concluded that the reduction activities of the samples were in the order of VNE-AgNPs > VNE > C-AgNPs. At the same time, C-AgNPs did not show any activity.

## Conclusion

In the present study, the phytochemical profile and chemical and biological properties of *Verbascum nudatum* species were investigated for the first time. There are very few studies on nanoparticle synthesis via *Verbascum* species. In this respect, *Verbascum* spp. was used to synthesize AgNPs for the first time, and the properties of the obtained nanoparticles were compared with those of C-AgNPs synthesized by chemical method. The results showed that the particle size of VNE-AgNPs was smaller (below < 30 nm), and therefore, the anticarcinogenic activity was higher in A549 cells. It was concluded that VNE-AgNPs increased inflammation by increasing TNF-α levels (333.45 ± 67.20 ng/mg-protein), which is a proinflammatory cytokine, and increased oxidative stress (compared to C-AgNPs) by increasing OSI levels (139.37 ± 12.04) in A549 cells. It was concluded that the increase in inflammation, oxidative stress, and cytotoxic effects of VNE-AgNPs may be related to the particle size of the nanoparticles and the bioactive components retained on the nanoparticle surface due to green synthesis. When the photocatalysis results were evaluated, it was determined that the effect of the substances used in the study on MB degradation was in the order of VNE-AgNPs > VNE > C-AgNPs. These data suggest that VNE-AgNPs prepared using *Verbascum nudatum* aqueous extract can be a potential catalyst to reduce the environmental damage of organic pollutants. However, to say this clearly, it is recommended to investigate the photocatalytic reactions of VNE-AgNPs with a much different structure and number of organic pollutants.

### Supplementary Information

Below is the link to the electronic supplementary material.

Supplementary file1 (DOCX 2323 KB)

## Data Availability

Data will be made available on request.
