# Supplementary material for: Environmentally friendly silver nanoparticles synthesized from Verbascum nudatum var. extract and evaluation of its versatile biological properties and dye degradation activity
Source: Environ Sci Pollut Res Int. 2024 Apr 29;31(23):33482–94. doi: 10.1007/s11356-024-33424-5 (PMC11136752; doi:10.1007/s11356-024-33424-5)
Supplement: Supplementary file 1 — Supplementary file1 (DOCX 2323 KB) [file 11356_2024_33424_MOESM1_ESM.docx]

**Environmentally Friendly Silver Nanoparticles Synthesized from *Verbascum nudatum var* extract and evaluation of its versatile biological properties and dye degradation activity**

**Ömer Hazman ^1,2^, Gofur Khamidov^2^, Mustafa Abdullah Yilmaz^3,4^, Mehmet Fatih Bozkurt^5^, Mustafa Kargioğlu^6^, Davlat Tukhtaev^2^, Ibrahim Erol^1,7*^**

^1^Afyon Kocatepe University, Faculty of Science and Arts, Department of Chemistry, 03200 Afyonkarahisar, Türkiye.

^2^Samarkand State University, Institute of Biochemistry, Department of Organic Synthesis and Bioorganic Chemistry, University blvd-15, Samarkand, Uzbekistan.

^3^Dicle University, Science and Technology Research and Application Center, 21280, Diyarbakır, Türkiye.

^4^Dicle University, Faculty of Pharmacy, Department of Analytical Chemistry, 21280, Diyarbakır, Türkiye.

^5^Afyon Kocatepe University, Faculty of Veterinary Medicine, 03200 Afyonkarahisar, Türkiye.

^6^Afyon Kocatepe University, Faculty of Science and Arts, Molecular Biology and Genetics, 03200 Afyonkarahisar, Türkiye.

^7^Samarkand State University, Institute of Biochemistry, [Department of Polymer Chemistry and Chemical Technology](https://samdu.uz/en/pages/polimerlar_kimyosi_va_kimyoviy_texnologiya), University blvd-15, Samarkand, Uzbekistan.

^*^Corresponding author. Tel.: +90-272-2182337; Fax: +90-272-2281422; Email address: [ierol@aku.edu.tr](mailto:cifcicemal@aku.edu.tr) (I. Erol).

**Materials and methods**

**Materials**

The specimens of Verbascum nudatum var. nudatum used in the study were collected on 22.06.2021 (within the flowering period of the species) from the vicinity of Çığrı village (Altitude: 1229 m, GPS: 36 S 0241720, UTM 4190691), Başmakçı district, Afyonkarahisar province, Turkey. The plant was collected from its natural habitat and taxonomically identified by Prof. Dr. Mustafa Kargıoğlu from the Department of Molecular Biology, Afyon Kocatepe University. The collected plants were rinsed in purified water and allowed to dry in a dark place at room temperature. The species was included in Afyon Kocatepe University herbarium (herbarium no: Kargıoğlu 10777). "Flora of Turkey" was used to identify the species.

**Plant material and extraction**

Dried *Verbascum nudatum* flowers were prepared for extraction along with the branch. The samples were broken into small pieces by hand and ground using a blender (Waring 32BL80). In this way, 10 gr of the plant transformed into homogenized plant powder form. It was weighed and 100 mL of deionized water (1:10 w/v ratio) was added. It was incubated in a glass bottle on a heated magnetic stirrer at 60 ⁰C, 750 rpm for 2 hours. Then, the ultrasonic water bath was set to 60 ⁰C and 35 kHz operating frequency. The herb solvent mixtures in glass bottles were kept in a water bath for 2 hours. After the solutions were removed from the water bath, they were kept in the dark at room temperature for 24 hours. The prepared extracts were filtered through filter paper (Whatman, Grade 589/1) to remove plant parts and collected in a separate flask. The aqueous extract mixture was placed in 15 mL falcon tubes. Components in the extract that were very small and could not be separated from the aqueous extract by filtration were precipitated by centrifugation (5 minutes, 8500 rpm). The aqueous solution of *Verbascum nudatum* branch-flower extract (VNE) obtained in this way was used in nanoparticle synthesis. The extract to be used in determining biological properties and phytochemical components was obtained by evaporating the solvent of the water-extract mixture. The solvent (water) of VNE was removed under a vacuum in an evaporator device (Heidolph, Germany). The obtained extracts were poured into glass petri dishes and allowed to dry for a few days at room temperature to better purify them from the solvents. The thoroughly dried VNE in the Petri dishes was scraped with a scraper and put into small glass bottles. The extracts were stored at +4 ⁰C until used in other laboratory analyses (such as total phenolic substance analysis, VNE component analysis, and analyzes related to antioxidant/oxidant parameters).

**Preparation of AgNPs**

In producing *Verbascum nudatum*-silver nanoparticles (VNE-AgNPs), the green method (Sivrier et al. 2023; Artun et al. 2023) was used, considering plant extracts as reducing power. AgNO_3_ (Sigma) was preferred as the silver source in nanoparticle synthesis. 10 mM AgNO_3_ solution was prepared using ultrapure water (conductivity 18.2 MΩxcm). The prepared AgNO_3_ and the VNE, the preparation method of which was explained in the previous section, were taken in equal volumes and combined in a bottle. It was mixed at 700 rpm at 60 °C for 4-6 hours using a magnetic stirrer. At the end of the incubation, it was observed that the color of the mixture changed to khaki-green and particles (VNE-Ag NPs) precipitated at the bottom of the mixture. The resulting VNE-AgNPs were obtained with the help of centrifugation. For this purpose, the mixture containing VNE-AgNPs was placed in falcon tubes (15 mL) and centrifuged at 8500 rpm for 5 minutes. The supernatants in the tubes were discarded and 3 mL of deionized water was added to each tube. By pipetting, washing was performed to eliminate any impurities that could not fully adhere to the VNE-AgNPs in the base. VNE-AgNPs precipitate was obtained again by centrifugation under the same conditions. In this way, VNE-AgNPs were purified by washing five times in total. Finally, the VNE-AgNPs received at the bottom of the tubes were poured into glass Petri dishes containing 3 mL of deionized water and left to dry in an oven at 60 °C overnight. At the end of the incubation, silver mirror formation was observed at the bottom of the petri dish. The dried VNE-AgNPs were scraped with a plastic spatula and transferred to a stock container. The process of obtaining nanoparticles is illustrated in Scheme 1.

**
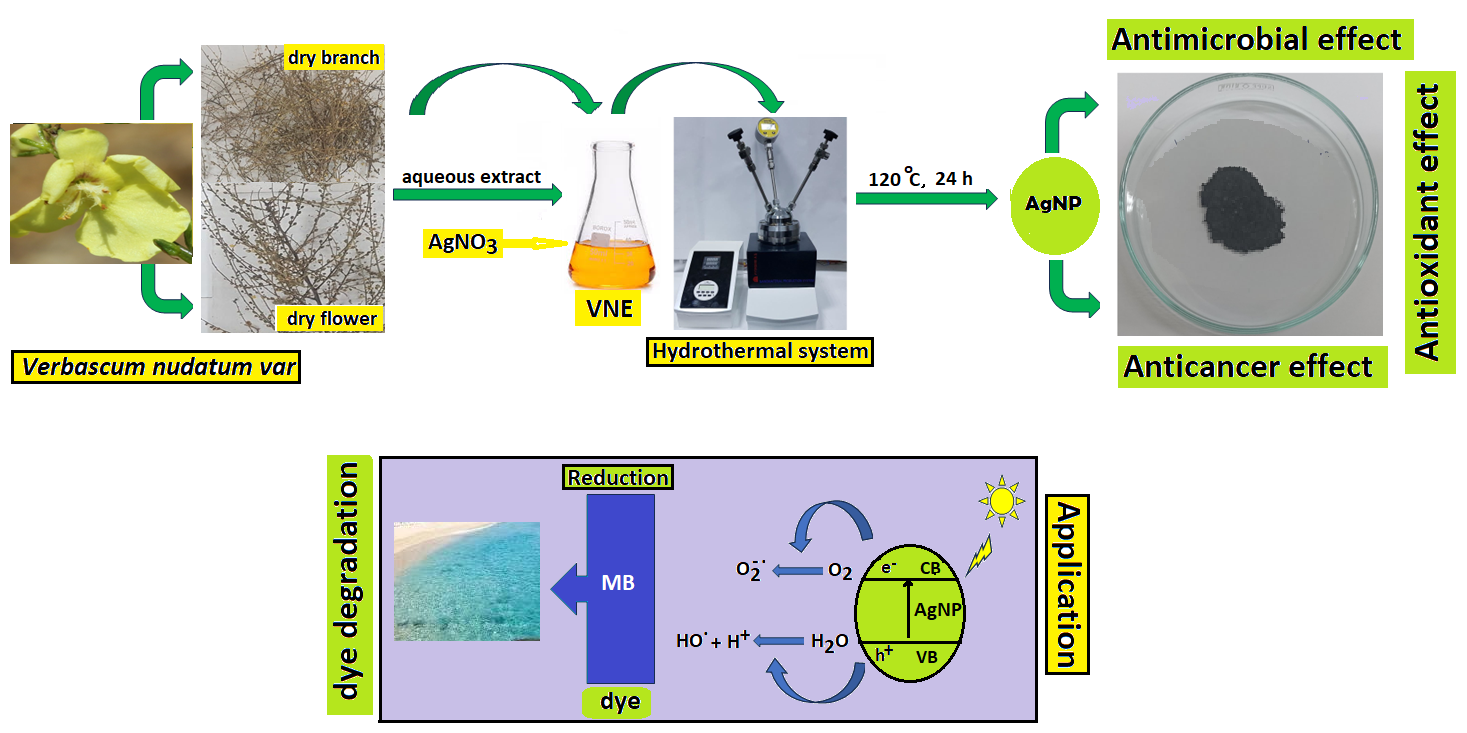
Scheme 1.** Preparation of the AgNPs from VNE.

**Determination of Chemical and Biological Properties**

In the present study, the qualitative and quantitative determination of the components of VNE and the total amount of phenolic substances were determined. Biological properties (oxidant/antioxidant activity, cytotoxicity levels, etc.) of VNE and nanoparticle (VIE-AgNPs, C-AgNPs) types were investigated. Thus, the chemical content, biological properties, and possible pharmacological activities of *Verbascum nudatum* species, which have not been studied in detail, were discussed. The photocatalytic capacity of VNE-AgNPs was investigated on the photodegradation of methylene blue (MB), a textile dye. On the other hand, the differences between the activities of AgNPs produced using VNE extract and C-AgNPs were tried to be determined. The methodology used for these purposes is presented in this section.

**Characterization**

The optical properties of the synthesized nanoparticles were determined using a UV-Vis absorption spectrophotometer (UV-1800, Shimadzu, Japan). Functional group analyses of the samples were determined using an FTIR spectrometer (Nicolet iS5 FTIR Spectrometer, Thermo Fisher, Germany) in the 4000-400 cm^-1^ range. The elemental percentage of the synthesized nanoparticles was determined using an energy-dispersive X-ray spectroscopy (XPS) (Philips EDAX/DX4). Field emission scanning electron microscopy (FESEM) (Hitachi Regulus 8230) and transmission electron microscopy (TEM) (JEOL JEM-1220) were used to determine the morphology of the samples. The crystallographic phases and structural properties of the synthesized nanoparticles were analyzed using X-ray diffraction (XRD). A Bruker D8 Advance diffractometer system with Cu Ka α radiation was used for XRD measurements. Measurements were performed with Cu Ka α radiation in the range 20°-80^o^ at a 2θ scan rate of 1.281 ^o^/min. A Shimadzu DTG-60H thermogravimetric analyzer was used to determine the thermal properties. Measurements were performed in an argon atmosphere at a heating rate of 20 ^o^C/min starting from the focal temperature up to 600 ^o^C. Dielectric measurements of AgNPs were performed with the Hioki LCR Meter impedance analyzer from 100 Hz to 2 kHz.

**Determination of Components in Extracts and Concentrations**

In the present study, a quantitative evaluation of 53 phytochemicals contained in VNE was performed. LC-ESI-MS/MS analysis system was used for this purpose. This system consisted of a Shimadzu-Nexera model ultra-high performance liquid chromatography (UHPLC) coupled with a tandem mass spectrometer. Analysis of the components was performed in a reversed-phase UHPLC system equipped with an autosampler (SIL-30AC model), a column oven (CTO-10ASvp model), a degasser (DGU-20A3R model), and dual pumps (LC-30AD model). Chromatographic conditions were optimized to ensure optimal separation of phytochemicals and to overcome suppression effects. A reversed-phase Agilent Poroshell 120 EC-C18 model (150 mm × 2.1 mm, 2.7 µm) analytical column was used for chromatographic separation. The obtained LC-ESI-MS/MS data were processed with LabSolutions software (Shimadzu). MRM (multiple reaction monitoring) mode was used to quantify phytochemicals (Yılmaz 2020). The 53 phytochemicals investigated in VNE content are as follows:Quercetin, Coumaric acid, Naringenin, Rosmarinic acid, Gallic acid, Apigenin, Quinic acid, Caffeic acid, Chlorogenic acid, Cyranoside, Protocatechuic acid, Rutin, Fumaric acid, Luteolin, Hesperidin, Isoquercitrin, Nicotiflorin, Coumarin, Gentisic acid, p-Cosmosiin, Astragalin, Quercitrin, Miquelianin, Protocatechuic aldehyde, Genistein, Hesperetin Chrysin, Acacetin, Epigallocatechin, Catechin, Tannic acid, Epigallocatechin gallate, Cynarin, 4-OH Benzoic acid, Epicatechin, Vanilic acid, Ferulic acid, Syringic acid, Vanillin, Syringic aldehyde, Daidzin, Epicatechin gallate, Piceid, Sinapic acid, Salicylic acid, o-Coumaric acid, Genistin, Ellagic acid, Fisetin, Daidzein,, Amentoflavone.

**Determination of Total Phenolic Substance Content of VNE**

The total phenolic content of caffeic acid, which will be used to compare the results of VNE and VNE extracts, was determined spectrophotometrically (Shimadzu, UV-1700) by measuring the absorbance at 765 nm using the Folin-Ciocalteu method described by Balkır et al (Balkır et al. 2023). A calibration curve was prepared using known amounts of gallic acid as a standard in the analyses. Total phenolic substance levels in VNE were calculated as Gallic Acid Equivalent (GAE) using the line equation obtained from sample absorbances and calibration curves. The results obtained were expressed as µgGAE/mg-extract.

**Evaluation of Biological Properties**

**Determination of DPPH Radical Scavenging Activity**

DPPH radical scavenging activity of the samples prepared with VNE, VNE-AgNPs, and C-AgNPs (Sigma, 576832) used in the study was determined by modifying the microwell method Khamidov et al. 2023). The stock DPPH solution prepared in methanol at 12 µM was used in the analyses. To compare the DPPH inhibition levels of the samples, DPPH inhibition levels of butylated hydroxytoluene (BHT), an antioxidant, were also determined. Aqueous dispersed mixtures of 0.1 mg/mL, 0.5 mg/mL, 1 mg/mL, 5 mg/mL, and 10 mg/mL concentrations of samples and BHT were prepared. In a 96-well microwell, 100 µL of samples at the determined concentrations were added to the pre-planned wells. DPPH solution was added to the control wells, and methanol was added to the blank well. DPPH solution with a concentration of 10 µM in a volume of 100 µL was added to the wells. The microwell was covered airtight and incubated at 37 ⁰C for 30 min on a horizontal shaker (300 rpm). At the end of incubation, the absorbance value of each well was determined at 540 nm (BioTek, ELx800). Absorbances and DPPH inhibition levels were calculated using the following formula.

DPPH inhibition (%) = [(Absorbance_blank_ – Absorbance_sample_) / (Absorbance_blank_)] x 100

**Determination of Antimicrobial Activity**

Antimicrobial activity levels of the substances used in the study (VNE, VNE-AgNPs and C-AgNPs) were determined by disc diffusion method (Bauer et al. 1966). The studies used two bacteria (*S. aureus* and *E. coli*) and one yeast (*C. albicans*) reference strain. One day after the microorganism strains were revived, inoculums were prepared according to 0.5 McFarland turbidity standard (10^6^-10^7^ cfu/mL). Mueller-Hilton agar (Merck) prepared under sterile conditions was added to clean petri dishes (9 mm diameter). After the gelation, an inoculum of microorganisms was added in a volume of 750 µL and spread on the agar. Three 6 mm diameter sterile blank discs (one for VNE, one for VNE-AgNPs, and one for C-AgNPs) and one antibiotic (penicillin 5 IU) were placed in each petri dish. A 10 µL volume of the sample mixture containing 20 µg of the active substance was applied to the pre-located discs. Petri dishes were incubated at 37 ^o^C for 24 hours. At the end of incubation, the inhibition zones formed around the discs were measured in mm units using a digital caliper.

**Determination of Cytotoxicity and Anticarcinogenic Activity**

3-[4,5-dimethylthiazole-2-yl]-2,5-diphenyltetrazolium bromide (MTT) assay was used to determine the cytotoxic/anticarcinogenic activity of VNE, VNE-AgNPs and C-AgNPs. Mouse fibroblast (L929) cells were used as a healthy cell line, and lung cancer (A549) cells were used as a cancer cell line.

**MTT Analysis**

Cells (L929 and A549 cells) were grown in high glucose Dulbecco's Modified Eagle Medium (DMEM) containing 10% fetal calf serum (Capricorn), 1% penicillin-streptomycin (Sigma), 1% glutamine (Sigma). After the cells were treated under sterile conditions, all incubations were performed in a CO_2_ incubator (Nüve) to provide 37 ⁰C, 5% CO_2_, and sufficient humidity. Cells were seeded in 96 well plates with 10^4^ cells in each well. After incubating the cells overnight and reaching 60-70% confluence rate, pre-prepared solutions of the materials used in the study (VNE, VNE-AgNPs, and C-AgNPs) at different concentrations (seven different concentrations in the range of 5-800 µg/mL) were applied at least three times. The same medium volume was added to the cells planned as a control group. After treatment, the cells were incubated for 24 hours. MTT salt was dissolved in phosphate buffer saline (PBS) at a 5 mg/mL concentration. 25 µL of the prepared MTT solution was added to each well. Cells were incubated for 3 hours. At the end of incubation, the medium in the wells was aspirated. Then, 200 µL dimethyl sulphoxide (DMSO) was added to each well. The formosan crystals formed by the living cells and MTT salt at the bottom of the well were dissolved in DMSO. The absorbance of the sample in each well was measured at 540 nm using an ELISA microplate reader (Biotek, ELx800). The cell viability in the absorbance of the control group to which only the medium was added was accepted as 100%, and the effect of each dose was calculated by the following equation.

Cell viabilty (%) = [(Absorbance_sample_) / (Absorbance_control_)] x 100

**Determination of oxidative stress and inflammation effects**

The effects of the samples on oxidative stress and inflammation were tested on A549 cells. For this purpose, VNE, VNE-AgNPs, and C-AgNPs were applied at 50 µg/mL concentrations to A549 cells that proliferated sufficiently in 75 cm^2^ flasks. Before application, dispersed mixtures were subjected to sonication and pipetting. 24 hours after the applications, the cells were removed from the bottom of the flask by trypsinization-detrypsinization and formed into a cell pellet on the bottom of a 15-millimeter falcon. Lysis buffer (500 µL) prepared in PBS (pH:7.4) containing 50 mM HEPES buffer, 1% triton-x100, and 8% protease inhibitor cocktail was added to the cell pellet. After pipetting and vortexing, the mixture was centrifuged at 8500 rpm and +4 ⁰C. Parameters related to oxidative stress and inflammation were analyzed in the resulting lysates. Information about the four experimental groups created to carry out these analyses is presented in the Table 1 below.

**Table 1.** Experimental groups and applications designed to determine oxidative stress and inflammation

| **Experimental groups*** | **Applications to cells** |
| --- | --- |
| **Control** | The same medium volume was added as the active ingredients were applied to the other groups. |
| **VNE** | VNE solution was added to a final concentration of 50 µg/mL. |
| **VNE-AgNPs** | VNE-AgNPs disperse mixture was added to a 50 µg/mL final concentration. |
| **C- AgNPs** | C-AgNPs disperse mixture was added to a final concentration of 50 µg/mL. |

* Applications in the experimental groups were performed 3 times. The samples (cells) obtained after the applications were used in biochemical and immunocytochemical analyses.

Using total antioxidant capacity (TOC) and total antioxidant capacity (TAC) values determined with commercial kits (Rell Assay, Turkey), OSI values of the samples were calculated with the formula OSI=TOC/TAC. TNF-α, IL-1β and β-Defensin levels in cell lysates were analyzed using human-specific commercial ELISA kits (eLabscience) to determine inflammation levels. Oxidative stress and inflammatory parameter levels determined using cell lysate were normalized by dividing by the total protein value of each sample. The total protein values of the samples were determined using a microplate (BioTek, ELx800) reader at 590 nm in a 96-well plate, using a method modified from the Bradford method (Bradford 1976). Bovine albumin was used as the standard in total protein analyses, and Coomassie Brilliant Blue G-250 (sigma) was used as the reagent.

**Immunocytochemical Analysis**

The collected cells were fixed in 10% buffered neutral formaldehyde solution for 36 hours. They were washed 5 times with distilled water, made into pellets, and embedded in paraffin blocks. The blocks were cut to 5 μm thickness and placed on adhesive slides. The samples were stained with immunohistochemical (Hazman et al. 2021). For this purpose, rabbit monoclonal anti Ki-67 (1/100 dilution, Thermo-Scientific, RM-9106, USA) and rabbit polyclonal anti iNOS (1/1000 dilution, Thermo-Pierce PA3-030A, USA) antibodies were dropped onto the samples. Biotinized Anti-rabbit antibody (1/100 dilution, anti-rabbit (IgG BA110, Vector Laboratories Inc., CA, USA)) was secondarily applied and incubated for 30 minutes. After washing, it was treated with Peroxidase enzyme-conjugated streptavidin (Standard Vectastain Elite ABC Kit, PK-6100, Vector Laboratories Inc, CA, USA) for 30 minutes. Washed with TBS. Finally, the reaction was colored by applying the peroxidase substrate 3-amino-9-ethylcarbazole (AEC). Gill's (III) hematoxylin was used for the base, and the slides were closed with an aqueous mounting medium. All samples were examined under a light microscope (Zeiss Lab-A1 / Axiocam-ICC5 imaging system) and analyzed using image J software.

**Photocatalytic activity**

The photocatalytic performance of VNE-AgNPs was tested on methylene blue (MB) dye compared with C-AgNPs (Chinnasamy et al. 2023). In determining the environmental effects of nanoparticles, the experimental model in which photocatalytic methylene blue (MB) degradation levels are used is frequently used in the literature. These methods were modified and used in our study. For this purpose, MB solution (20 mg/L), which is preferred as the model pollutant, was prepared in deionized water. VNE, VNE-AgNPs and C-AgNPs used in our study were weighed as 50 mg and placed in petri dishes. Nothing was added to the final petri dish used as the control group. Petri dishes were exposed to UV-A rays provided by four parallel fluorescent lamps (Philips, 8 W each lamp) emitting at 365 nm for three hours. The distance of these lamps to the petri dishes was 25 cm. The average value of the radiation energy reaching the Petri dishes determined using a radiometer (Delta Ohm, DO9721), was 17 Wm^-2^ in the range of 315-400 nm. During irradiation, dye solution samples were taken from the system at 20 minute intervals, and the absorbance values of MB at 663 nm were determined with a UV-vis spectrophotometer (Shimadzu UV1800). The effect of nanoparticles and VNE on MB degradation was calculated using the formula below.

Methylene Blue Degradation (%) = [100 - (Absorbance_sample_ / Absorbance_control_) x 100]

**Statistical Method**

Data from analyses with at least three repetitions were given as "mean standard deviation." Statistical evaluation of the data was performed using SPSS20 software. The presence/absence of statistical differences between the data/groups was determined using a one-way Analysis of variance (ANOVA) test, and the differences between the data/groups were determined using the Duncan post hoc test.


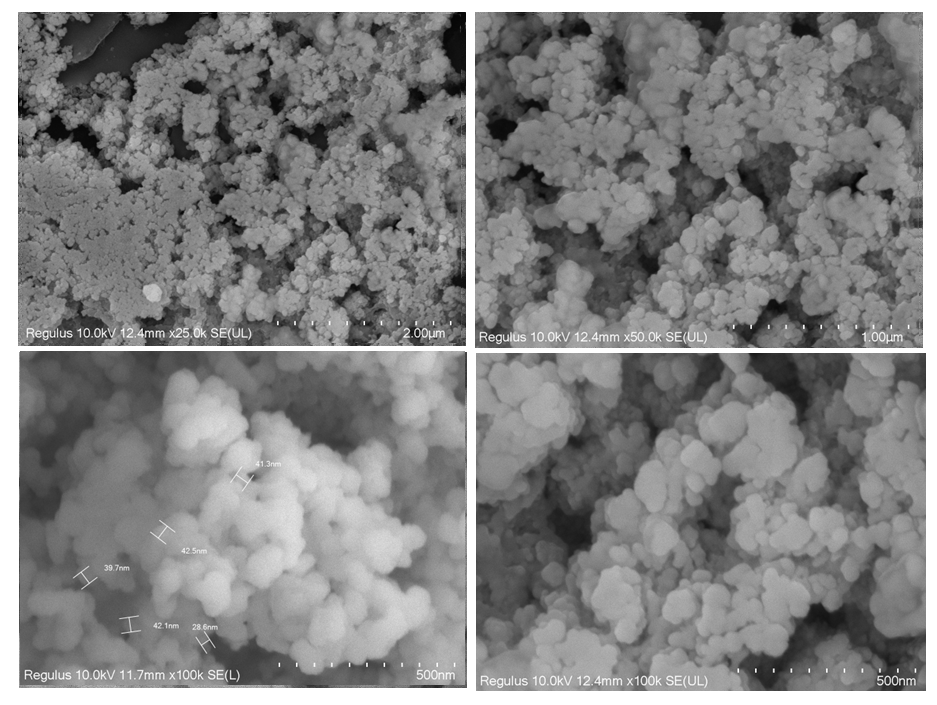


**Fig. S1** FESEM image of the VIE-AgNPs.


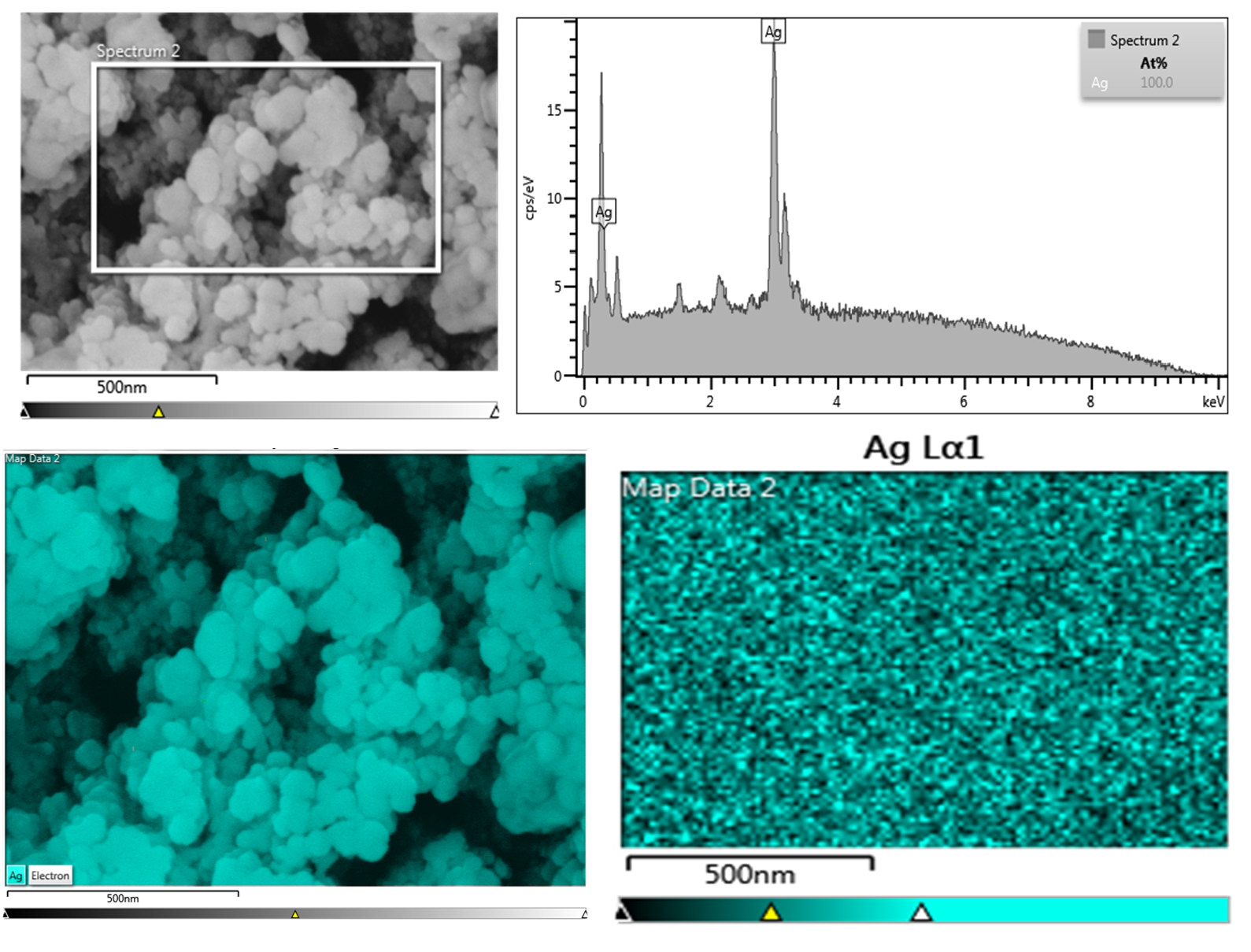


**Fig. S2** EDS analysis of the VIE-AgNPs.


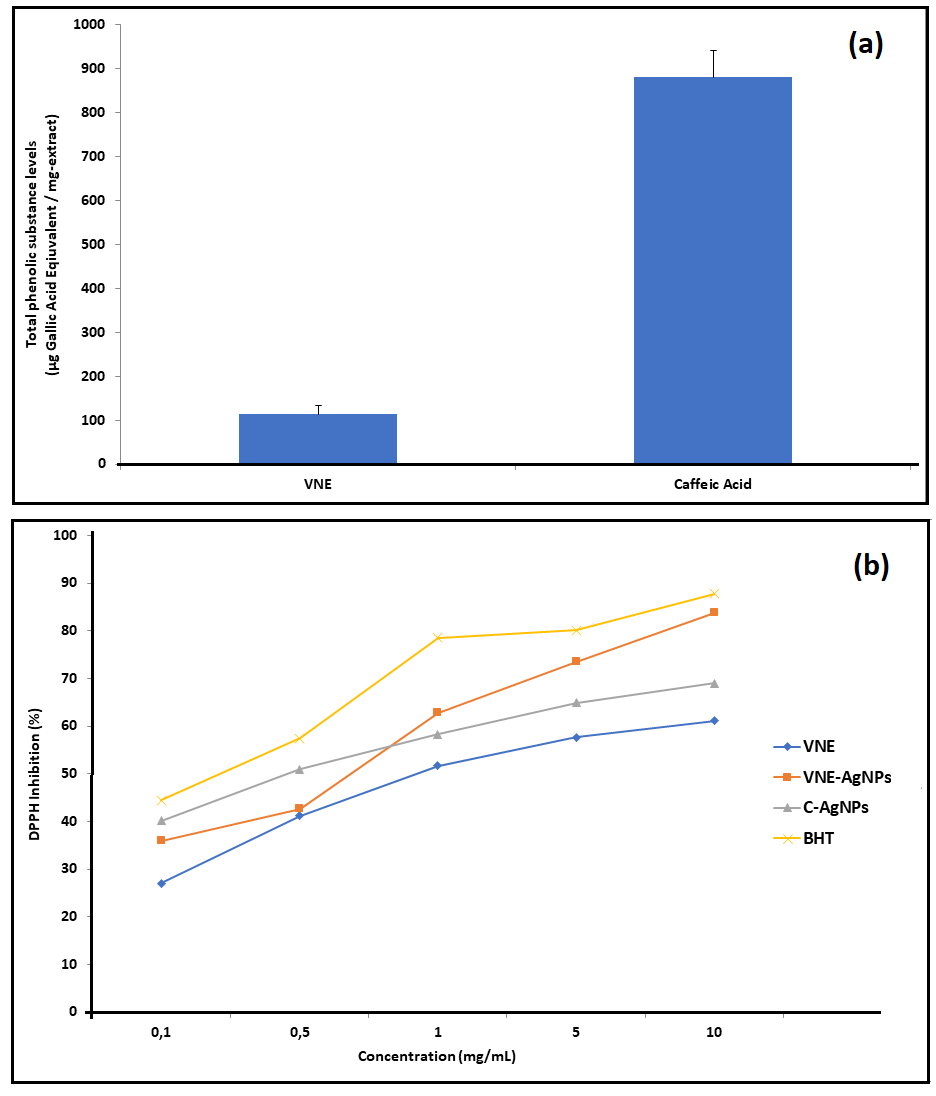


**Fig. S3** (a) Total amount of phenolic substances in *Verbascum nudatum* extract, (b) DPPH inhibition levels of VNE and Nanoparticles.


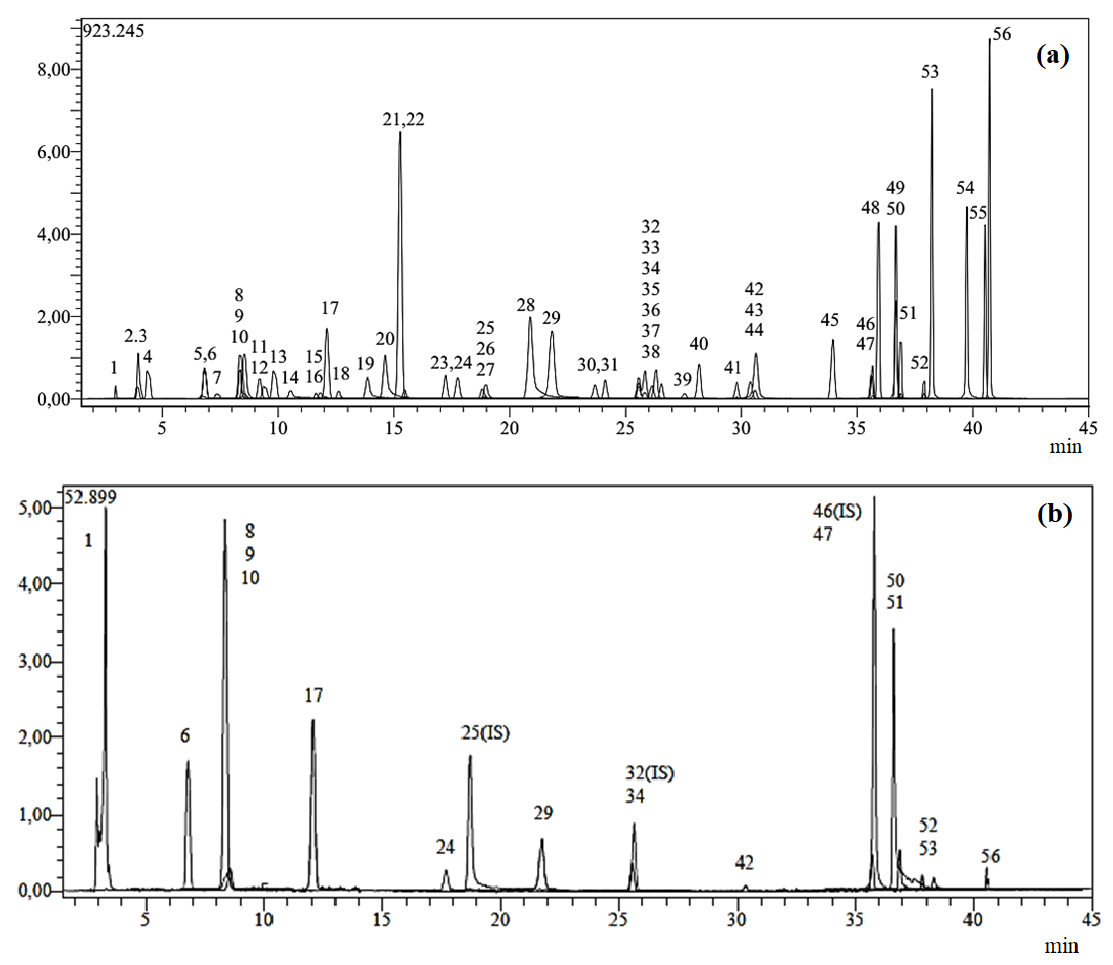


**Fig. S4** LC-MS/MS chromatograms used for qualitative and quantitative determination of the components in the VNE.

a; Chromatogram of standards; 56 peaks were identified in the standard chromatogram. Of these peaks, 53 belonged to the standards whose presence in the extracts was investigated qualitatively/quantitatively, and 3 (Ferulic acid-D3-ISh, Rutin-D3-ISh, 46; Quercetin-D3-ISh) belonged to the standards used to determine the optimization of the instrument operation.

b; Chromatogram of VNE; the peaks of 16 components detected in the VNE are included in the chromatogram.

**References**

Artun H, Hazman Ö, Tillayev S, Erol I (2023) Preparation of nanocomposite based on chitosan-

PDCOEMA containing biosynthesized ZnO: Biological and thermal characterization. Int J Biol Macromol 242:124753. [https://doi.org/10.1016/j.ijbiom ac.2023.124753](https://doi.org/10.1016/j.ijbiom%20ac.2023.124753)

Balkır Ş, Hazman Ö, Aksoy L, Yılmaz MA, Çakır O, Kara R, Erol I(2023). Phytochemical

profile, antioxidant and antimicrobial potency of aerial parts of Salvia tomentosa Miller. Acta Chim Slov 70(2), 218-225. [https://doi.org/10.17344/acsi.2023. 8008](https://doi.org/10.17344/acsi.2023.%208008)

Bauer AW, Kirby WM, Sherris JC, Turck M (1966) Antibiotic susceptibility testing by standard

single disc diffusion method. Am J Clin Pathol 45:493–496. <https://doi.org/>10.1093/ ajcp/45.4_ts.493

Bradford MM, (1976) A rapid and sensitive method for the quantitation of microgram

quantities of protein utilizing the principle of protein-dye binding. Anal Biochem 72, 248–254. <https://doi.org/10.1016/0003-2697(76)90527-3>

Chinnasamy R, Chinnaperumal K, Venkatesan M, Jogikalmat K, Cherian T, Willie P, Malafaia

G (2023) Eco-friendly synthesis of Ag-NPs using Endostemon viscosus (Lamiaceae): Antibacterial, antioxidant, larvicidal, photocatalytic dye degradation activity and toxicity in zebrafish embryos. Environ Res 218, 114946. [https://doi.org/ 10.1016/j.envres.2022.114946](https://doi.org/%2010.1016/j.envres.2022.114946).

Hazman Ö, Sarıova A, Bozkurt MF, Ciğerci İH (2021) The anticarcinogen activity of β-arbutin

on MCF-7 cells: Stimulation of apoptosis through estrogen receptor-α signal pathway, inflammation and genotoxicity. Mol Cell Biochem 476(1):349-360. https: //doi.org/10.1007/s11010-020-03911-7

Khamidov G, Hazman Ö, Erol I. (2023) Thermal and biological properties of novel sodium

carboxymethylcellulose-PPFMA nanocomposites containing biosynthesized Ag-ZnO hybrid filler. Int J Biol Macromol 257, 128447. <https://doi.org/10.1016/j>. ijbiomac.2023.128447

Sivrier M, Hazman Ö, Tillayev S, Erol I (2023) Novel Bionanocomposites Containing Green

Synthesized Silver NPs of a Carboxymethyl Cellulose-Based Blend; Thermal, Optical, Biological and Dielectric Properties. J Polym Environ 31(9) 3857-3874. https://doi.org/[10.1007/s10924-023-02866-2](http://dx.doi.org/10.1007/s10924-023-02866-2)

Yilmaz MA, (2020) Simultaneous quantitative screening of 53 phytochemicals in 33 species of

medicinal and aromatic plants: A detailed, robust and comprehensive LC–MS/MS method validation. Ind Crops Prod 149, 112347. [https://doi.org/10.1016/j. indcrop.2020.112347](https://doi.org/10.1016/j.%20indcrop.2020.112347)
